# Outcome in hip fracture patients related to anemia at admission and allogeneic blood transfusion: an analysis of 1262 surgically treated patients

**DOI:** 10.1186/1471-2474-12-262

**Published:** 2011-11-21

**Authors:** Anne JH Vochteloo, Boudewijn LS Borger van der Burg, Bart JA Mertens, Arthur HP Niggebrugge, Mark R de Vries, Wim E Tuinebreijer, Rolf M Bloem, Rob GHH Nelissen, Peter Pilot

**Affiliations:** 1 Department of Orthopaedics, Reinier de Graaf Group, PO Box 5011 2600 GA Delft, the Netherlands; 2 Department of Orthopaedics Leiden University Medical Center, PO Box 9600, 2300 RC Leiden, the Netherlands; 3 Department of Surgery, Rijnland Hospital, PO Box 4220, 2350 CC Leiderdorp, the Netherlands; 4 Department of Medical Statistics and Bioinformatics, Leiden University Medical Center, PO Box 9600, 2300 RC Leiden, the Netherlands; 5 Department of Surgery, Bronovo Hospital, PO Box 96900, 2509 JH The Hague, the Netherlands; 6 Department of Surgery, Reinier de Graaf Group, PO Box 5011 2600 GA Delft, the Netherlands; 7 Department of Surgery-Traumatology, Erasmus MC, University Medical Center, PO box 2040 3000 CA Rotterdam, the Netherlands

## Abstract

**Background:**

Anemia is more often seen in older patients. As the mean age of hip fracture patients is rising, anemia is common in this population. Allogeneic blood transfusion (ABT) and anemia have been pointed out as possible risk factors for poorer outcome in hip fracture patients.

**Methods:**

In the timeframe 2005-2010, 1262 admissions for surgical treatment of a hip fracture in patients aged 65 years and older were recorded. Registration was prospective from 2008 on. Anemic and non-anemic patients (based on hemoglobin level at admission) were compared regarding clinical characteristics, mortality, delirium incidence, LOS, discharge to a nursing home and the 90-day readmission rate. Receiving an ABT, age, gender, ASA classification, type of fracture and anesthesia were used as possible confounders in multivariable regression analysis.

**Results:**

The prevalence of anemia and the rate of ABT both were 42.5%. Anemic patients were more likely to be older and men and had more often a trochanteric fracture, a higher ASA score and received more often an ABT. In univariate analysis, the 3- and 12-month mortality rate, delirium incidence and discharge to a nursing home rate were significantly worse in preoperatively anemic patients.

In multivariable regression analysis, anemia at admission was a significant risk factor for discharge to a nursing home and readmission < 90 days, but not for mortality. Indication for ABT, age and ASA classification were independent risk factors for mortality at all moments, only the mortality rate for the 3-12 month interval was not influenced by ABT. An indication for an ABT was the largest negative contributor to a longer LOS (OR 2.26, 95% CI 1.73-2.94) and the second largest for delirium (OR 1.67, 95% CI 1.28-2.20).

**Conclusions:**

This study has demonstrated that anemia at admission and postoperative anemia needing an ABT (PANT) were independent risk factors for worse outcome in hip fracture patients. In multivariable regression analysis, anemia as such had no effect on mortality, due to a rescue effect of PANT. In-hospital, 3- and 12-month mortality was negatively affected by PANT, with the main effect in the first 3 months postoperatively.

## Background

Due to the ageing of the Western population, the number of elderly people will increase. Although age-adjusted incidence of hip fractures in many countries, like the US and Sweden, declined in the population of 50 years and older, there is a right-shift in hip fracture distribution towards the highest age groups [1]. These old patients have several co-morbidities, often described as the "frailty syndrome" [2]. One of the typical features of this syndrome is an anemic status [2]. Increasing evidence suggests that low hemoglobin concentration is common in elderly patients and adversely affects morbidity and mortality, especially if they are in need of surgery [2-5].

Several studies on anemia in hip fracture patients reported on increased morbidity, prolonged admission, higher readmission rate and increased mortality rate [6-8]. Studies on allogeneic blood transfusion (ABT) in hip fracture patients show ambivalent findings regarding outcome and mortality [9-14].

In this study, the effect of preoperative hemoglobin level and perioperative ABT was evaluated in a large cohort follow-up study of 1262 admissions for surgical treatment for hip fracture in patients of 65 years and older. The focus of our analysis was on mortality, incidence of delirium, length of hospital stay, discharge to a nursing home and the 90-day readmission rate.

## Methods

An observational cohort study was conducted on hip fracture patients admitted to a 350- and a 450-bed teaching hospital in The Hague and Delft.

### Aim of the study

To determine whether anemia at admission and perioperative allogeneic blood transfusion (ABT) have an independent negative effect with mortality, occurrence of a delirium, length of hospital stay (LOS), discharge to a nursing home and 90-day readmission rate in surgically treated hip fracture patients, aged 65 years and older. Delirium has been chosen, as it is one of the most serious and most common major in-hospital complications.

### Patients and methods

All patients were extracted from our digital database that contains all consecutive hip fracture patients, admitted from January 2005 to January 2010. Data were collected retrospectively for patients in the timeframe 2005-2007 and prospectively for those in the period 2008-2009.

The exclusion criteria for this study were: age < 65 years, a pathologic hip fracture, a high-energy injury and conservative treatment. Duration of follow-up in all patients was 12 months. This resulted in a cohort of 1262 admissions for a hip fracture, in 1222 patients.

From the hospital's records (both digital and paper files), age, gender, ASA classification, type of fracture, type of treatment, type of anesthesia, pre- and postoperative hemoglobin level, perioperative need for an ABT, occurrence of a delirium, LOS and discharge location were collected onto a case record form (CRF) [15].

Diagnosis of delirium was based on criteria of the DSM IV [16]. Signs of a delirium are recorded in the medical and nursing records as a standard part of documentation of the daily characteristics of a patient. Delirium incidence in this series was scored based on these medical and nursing staff records. To calculate the 90-day readmission rate, all readmissions within 90 days after discharge were extracted from the digital hospital admission registration system. Postoperative mortality has been documented meticulously by repeated consultation of the population registers of the counties in the region of both hospitals as well as the hospital's patient registration systems. If present, readmissions and date of death were recorded at the CRF. As of 2008, all data were recorded prospectively in the CRF. All fore mentioned data were complete, both in the retrospective and prospective series and was performed by authors AV, BB, PP and AN.

Approval from the local ethical committee was not obtained, as this is an observational study without an intervention. Therefore, it is an evaluation of usual care as a part of good clinical practice. Since data could not be traced back to the individual patient there were no privacy issues.

### Anemia and ABT policy

In all patients the hemoglobin level was measured at admission. Anemia at admission was defined based on the criteria of the World Health Organization (WHO) [17]. These criteria classify anemia as a hemoglobin level below 7.5 mmol/L (12 g/dL) in women and below 8.1 mmol/L (13 g/dL) in men. They were tested and found sufficient for elderly by Izaks et al in their study on mortality in elderly patients [18].

In the Netherlands, the national CBO guideline advises ABT for subjects aged > 60 years when hemoglobin level drops below 5.0 mmol/L (8.0 g/dL) in the general population or 6.0 mmol/L (9.7 g/dL) if the patient has a serious cardiac condition or when anemia becomes symptomatic [19]. This guideline is general practice in both hospitals. Hemoglobin level was measured on the first postoperative day as a routine and if necessary repeated thereafter based on clinical judgment.

### Statistical analysis

Demographic continuous data are presented as means, with standard deviations (SD). Categorical data are presented as the number of subjects in the category, along with the percentages. Chi-square test and Fisher's exact test were used for comparing groups of categorical data.

The cohort was compared regarding differences in clinical characteristics and outcome between the anemic and non-anemic patients, based upon hemoglobin level at admission.

Univariate analysis performed to test the association between anemia and mortality (in-hospital, 3- and 12-month), delirium, LOS categories (≤ versus > 11 days), discharge to a nursing home and 90-day readmission rate. Separate logistic regression analyses were executed for each outcome, for the mortality Cox proportional hazards regression analysis was used to calculate the hazard ratio and 95% confidence interval. In these analyses, correction was performed for the possible confounders; age, gender, perioperative risk (ASA classification I/II or III/IV), receiving an ABT, type of fracture (neck of femur, (inter-) trochanteric or subtrochanteric), and type of anesthesia (general or spinal). Because of the clinical relation between the anemia and receiving an ABT collinearity diagnostics of the logistic regression models were examined.

To robustify the analysis for LOS, this parameter was changed into a binary summary outcome, i.e. ≤ or > 11 days (the median), as the distribution of the value LOS was very wide.

Patients classified ASA I or II and III or IV were combined to two groups, as the separate groups of ASA I (n = 82) and ASA IV (n = 50) classified patients were too small to be analyzed separately.

P-values lower than 0.05 were considered statistically significant. Odds ratios are displayed with a 95% confidence interval if the p-value < 0.05. All data were analyzed in SPSS 17.0 (SPSS Inc. Chicago, USA).

## Results

### Clinical characteristics

1262 admissions for a surgical treatment of a hip fracture (1222 patients) were extracted from the database; 932 (73.9%) were female. Mean (SD) age was 83.6 (7.1) years and mean (SD) follow-up was 3.5 (1.4) years. Mean (SD) hemoglobin level at admission was 7.7 (1.0) mmol/l. According to the WHO criteria, 536 patients (42.5%) were anemic [17]. Transfusion of one or more unit(s) of allogeneic erythrocytes was given to 536 patients (42.5%), in the large majority (97.9%) postoperatively. Surgery within 24 hours after admission was performed in 84.4% of all patients. Overall mortality was 40.4% (510 of 1262 patients).

### Anemic versus non-anemic patients

The patients were divided in an anemic and non-anemic group, based on the hemoglobin level at admission; clinical characteristics of the two populations are shown in Table 1.

**Table 1:** Relative risks for different demographic characteristics in anemic and non-anemic patients

Outcome	Anemic	Non-anemic	RR (CI)	P-value
	*n = 536*	*n = 726*		
**mean age in years (SD)**	84.9 (6.9)	82.6 (7.0)	n/a	< 0.001
**mean follow-up in months (SD)**	42.7 (16.9)	42.2 (15.9)	n/a	0.578
**Male gender**	170 (31.7)	160 (22.0)	1.31 (1.15-1.50)	< 0.001
**ASA III/IV**	200 (37.3)	201 (27.7)	1.28 (1.12-1.45)	< 0.001
**Fracture type**				
*-neck of femur*	252 (47.0)	450 (62.0)		< 0.001*
*-(inter) trochanteric*	266 (49.6)	256 (35.3)	0.70 (0.62-0.80)	< 0.001**
*-subtrochanteric*	18 (3.4)	20 (2.8)		
**Spinal anesthesia**	494 (92.2)	684 (94.2)	0.84 (0.67-1.05)	0.149
**ABT**	309 (57.6)	153 (21.1)	2.36 (2.08-2.68)	< 0.001

Values are given as number (percentage), unless mentioned otherwise.

* P-value comparing 3 treatment groups, **RR and p-value comparing neck of femur with (inter-) trochanteric fractures.

RR = relative risk; CI = confidence interval; ASA = American Society of Anesthesiologists Physical Status classification, ABT = allogeneic blood transfusion.

The anemic group was significantly different from the non-anemic regarding age, gender, ASA, fracture distribution and ABT rate. There was no significant difference in mean follow-up. The relative risks for the different outcome parameters according to univariate analysis are presented in table 2. In this analysis, LOS and the in-hospital and 3-12 month interval mortality rate were not significantly different between both groups.

**Table 2:** Relative risks for different outcome measures in anemic and non-anemic patients

	Anemic	Non-anemic	RR (CI)	P-value
	*n = 536*	*n = 726*		
**Mortality**				
*In-hospital *	30 (5.6)	31 (4.3)	1.17 (0.90-1.52)	0.277
*3-month *	113 (21.1)	87 (12.0)	1.42 (1.23-1.64)	< 0.001
*3-12 month *	60 (11.2)	71 (9.8)	1.09 (0.89-1.33)	0.415
*12-month *	173 (32.3)	158 (21.8)	1.34 (1.18-1.53)	< 0.001
**Delirium**	162 (30.3)	155 (21.3)	1.29 (1.13-1.48)	< 0.001
**LOS > 11 days**	272 (50.7)	368 (50.7)	1.00 (0.88-1.14)	0.984
**Discharge to a NH**	277 (51.7)	285 (39.3)	1.33 (1.17-1.51)	< 0.001
**90-day readmission rate**	69 (12.9)	65 (9.0)	1.24 (1.04-1.49)	0.025

Values are given as number (percentage), RR = relative risk, CI = confidence interval, LOS = length of stay, NH = nursing home

The 90-day readmission rate was significantly higher in anemic patients. Readmission for an orthopaedic reason (surgical site infection, revision operation or a contralateral hip fracture) was necessary in 43.5% (30 of 69) of the anemic patients and in 36.9% (24 of 65) of the non-anemics.

### Multivariable logistic and Cox regression analysis

The Spearman's correlation coefficient between anemia and an ABT was low (0.38, p < 0.001) and the tolerance and VIF values indicated no multicollinearity.

Anemia at admission was not a significant independent risk factor for mortality at any moment in multivariable Cox regression analysis (table 3). Receiving an ABT was a significant independent risk factor for in-hospital, 3- and 12-month mortality, as were higher age and ASA classification in all patients (both anemic and non-anemic). However, receiving an ABT was not a risk factor for the mortality in the 3 to 12 month interval. Anemia was a significant independent risk factor for discharge to a nursing home and readmission within 90 days. The odds ratio for anemia in the analysis for LOS was 0.63 when adjusted for ABT and age. The latter two both had an independent negative impact on LOS (OR 2.12 and 1.06). This meant that anemic patients had a shorter stay in hospital than non-anemic patients, when they were younger and did not receive an ABT. For the outcome parameters besides mortality, i.e. discharge to a nursing home, delirium, 90-day readmission rate and a LOS > 11 days, age played a more important role than ASA classification. Gender and type of anesthesia were of little importance. Type of hip fracture was not a significant independent contributor to any of our outcome parameters in multivariable analysis.

**Table 3:** Risk factors with a significant HR for mortality and a significant OR for the other outcome parameters

Outcome variable	Risk factor	HR/OR	95% CI	P-value
Mortality*				
*in-hospital*	Age in years	1.07	1.02 to 1.11	0.002
	ASA	2.76	1.66 to 4.59	< 0.001
	ABT	1.88	1.12 to 3.14	0.017
*3-month *	Age in years	1.06	1.04 to 1.09	< 0.001
	ASA	1.94	1.47 to 2.57	< 0.001
	ABT	1.48	1.10 to 2.01	0.011
	Anemia	1.30	0.96 to 1.76	0.088
*3-12 month *	Age in years	1.05	1.02 to 1.08	< 0.001
	ASA	1.78	1.26 to 2.52	0.001
*12-month *	Age in years	1.06	1.04 to 1.08	< 0.001
	ASA	1.89	1.52 to 2.34	< 0.001
	ABT	1.42	1.13 to 1.77	0.002
**Delirium**	Age in years	1.06	1.04 to 1.08	< 0.001
	ASA	1.47	1.11 to 1.93	0.006
	ABT	1.67	1.28 to 2.20	< 0.001
	Male gender	1.99	1.49 to 2.66	< 0.001
**LOS > 11 days**	Anemia	0.63	0.48 to 0.81	< 0.001
	Age in years	1.07	1.05 to 1.08	< 0.001
	ABT	2.26	1.73 to 2.94	< 0.001
	ASA	1.24	0.97 to 1.59	0.089
**Discharge to a NH**	Anemia	1.41	1.10 to 1.81	0.007
	Age in years	1.05	1.04 to 1.07	< 0.001
	General anesthesia	1.67	1.05 to 2.64	0.029
	Male gender	0.75	0.58 to 0.99	0.038
	ABT	1.24	0.96 to 1.61	0.097
**90-day readmission rate**	Anemia	1.61	1.11 to 2.33	0.011
	Age in years	0.97	0.94 to 0.99	0.006
	ASA	1.43	0.99 to 2.09	0.060
	General anesthesia	0.35	0.13 to 0.99	0.047

Female gender, ASA classification I-II, having no anemia, receiving no allogeneic blood transfusion and spinal anesthesia are reference categories.

* Expressed in HR (Hazard Ratio), all other risk factors in OR (Odds Ratio)

CI = confidence interval, LOS = length of stay, NH = nursing home, ASA = American Society of Anesthesiologists Physical Status classification, ABT = allogeneic blood transfusion.

### Anemia effecting ABT and vice versa

Mortality in patients with anemia at admission (21.1%) was higher than in non-anemics (12%, p < 0.001) and mortality in all patients that received an ABT (22.7%) was higher compared to those that did not (11.9%, p < 0.001).

The in-hospital mortality rate of anemic patients that received an ABT (6.5%) compared to those who did not (4.4%), was comparable (p = 0.30). However, the mortality rates in preoperative non-anemic patients who received an ABT compared to those who did not were respectively 9.8% and 2.8% (p < 0.001).

The 3-month mortality in anemic patients was 24.3% in transfused and 16.7% in non-transfused patients (p = 0.035) and in non-anemic patients rates 19.6% and 9.9% (p = 0.001) in transfused and non-transfused patients respectively.

The relative risk (RR) for 3-month mortality in patients with anemia at admission is significantly increased in patients that did not receive an ABT (RR 1.49, CI 1.13-1.96, p = 0.07) this was not the case in those that did receive an ABT (RR 1.09, CI 0.95-1.26, p = 0.26).

**Figure 1:**
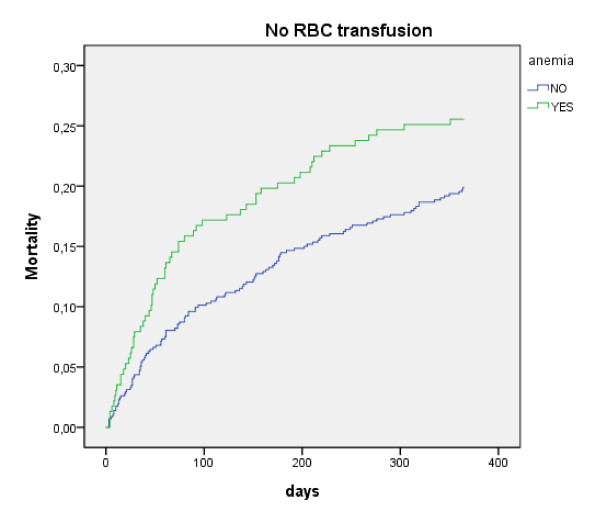
**mortality in patients that did not receive an allogeneic blood transfusion (ABT)**.

**Figure 2:**
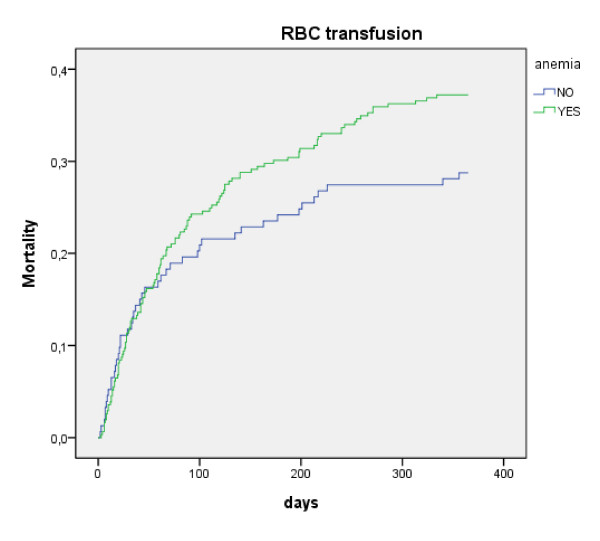
**mortality in patients that received an allogeneic blood transfusion (ABT)**.

As demonstrated in Figure 1 and 2, this effect of transfusion on mortality between anemic and non-anemic patients disappeared after about 3 months; the curves of anemic and non-anemic patients that receive an ABT (Figure 2) are parallel the first 90 days whereas the curves of those that do not receive an ABT diverge (Figure 1).

Mortality in patients that received an ABT was comparable in patients that were anemic at admission (24.3%) compared to the non-anemic (19.6%, p = 0.26).

In patients that did not receive an ABT mortality was higher in anemic (16.7%) than in non-anemics (9.9%, p = 0.07).

The RR for 3-month mortality in patients receiving an ABT was comparable between anemic patients (RR = 1.45, CI 1.02-2.06, p = 0.035) and non-anemic patients (1.97, CI 1.32-2.95, p = 0.001).

## Discussion

In the current study we demonstrate that receiving an ABT, an ASA classification III/IV and higher age were risk factors for postoperative mortality. In the same analysis, anemia at admission was a significant risk factor for discharge to a nursing home and readmission within 90 days, but not for a higher mortality. LOS in anemic patients, corrected for age and ABT, was shorter. Finally, receiving an ABT was correlated with a longer LOS and a higher delirium incidence.

### Anemic versus non-anemic patients

The incidence of anemia and the rate of ABTs in this cohort were consistent with previous studies in hip fracture patients [6-9,11-14].

Anemic patients were more likely to be older and to be men, to have a trochanteric fracture and a higher ASA score and to receive an ABT; the latter two results were demonstrated by others as well [7,8]. These studies [7,8] could not find a difference in gender distribution, one other could [6]. Others have shown that higher age, female gender and worse health status are correlated to intertrochanteric fractures [20-22]. These findings show the complex correlation between anemia as a mirror of a poor general condition (expressed in higher ASA scores), age and fracture type and gender

### Multivariable logistic and Cox regression analysis

We found anemia not to be a significant risk factor for mortality in Cox regression analysis, in contrast to several other studies [6-9]. However, the largest of these studies contained only 791 patients and some methodological concerns could be identified [6]. We consider anemia therefore to be more a proxy for frailty than a direct cause related to mortality.

Receiving an ABT was a risk factor for mortality at all follow-up moments, but not for the 3 to 12 months interval with its main effect in the first 3 months.

Mortality in transfused hip fracture patients was either higher or equal in previous series [10-14]. The two largest series were described by Johnston et al (n = 3571) and Carson et al (n = 8787) [12,14]. Johnston et al found a negative correlation at 120 and 365 days in univariate, but not in in multivariable analysis [12]. Carson et al found no effect on 30- and 90-day mortality [14]. In contrast, fewer complications were seen in restrictive ABT regimen patients who had total hip and knee replacements [23].

Age and ASA classification as risk factors for mortality and poor outcome do not need further explanation; the negative effect of the latter in hip fracture patients has recently been published [24].

Younger patients with anemia who received no ABT had a shorter LOS in our series. LOS is more often reported upon in studies on anemia than in those on ABTs [7-14]. Gruson, and Halm found a longer LOS in anemic patients, whereas Hagino did not [7-9]. The only study on ABT in hip fracture patients that reported LOS found no difference [10]. Younger age is most probably the reason for the shorter LOS, not anemia or receiving no ABT.

Receiving an ABT did and anemia did not have a negative effect on delirium incidence in this series. None of the studies on anemia and ABTs in hip fracture patients we found, reported delirium incidence. We would have expected anemia to be of negative influence as well, because higher age and ASA classification and male gender were negative contributors; all part of the the anemic patient profile. This is probably due to the fact that delirium occurs as a result of many more factors, like cognitive impairment and medication.

Finally, anemia at admission was of negative contribution to the 90-day readmission rate, ABT was not. Halm et al reported on readmissions, they found a lower 60-day readmission rate in patients with a higher hemoglobin level and in those receiving an ABT [9,13]. These different findings are probably due to the fact anemia and an ABT are only two minor of the many factors influencing the readmission rate.

### Anemia effecting ABT and vice versa

As demonstrated, receiving an ABT had a rescue effect on mortality in anemic patients in the first 3 months. This was probably partly due to correction of the negative effect of anemia at admission. The RR for 3-month mortality in patients receiving an ABT was comparable between anemic and non-anemic patients; both groups are negatively affected by an ABT.

## Limitations

Our study had a large sample size and the combined analysis of both anemia at admission and ABTs has never been done before. Limitations were that part of the data was collected retrospectively and documentation of occurrence of a delirium was based on clinical observations as described in the DSM-IV [16]. Although these criteria are sufficient, it is better to use a specific delirium score like the Confusion Assessment Score [25].

The time interval from fracture to admission is no of influence on the measured hemoglobin level at admission since in the Netherlands the mean interval between an accident and admission is no longer than a few hours.

Several limitations of the analysis for the impact of receiving an ABT can be identified. The number of ABTs was not reliably registered in the large majority of the patients, which made an interesting extra analysis using the number of transfusions not valid. For male and female patients different cut-off points for anemia (defined by the WHO) were used, but the transfusion threshold was not different [17,19]. Furthermore, the decision in daily clinical practice to give an ABT is not only based on the transfusion thresholds, but on clinical aspects as well as defined in the CBO guideline [19]. However, this represents daily practice for most hospitals.

Because of these limiting factors we will not presume a causal link between mortality and the other outcome parameters and receiving an ABT but we rather call it "postoperative anemia necessitating a transfusion, PANT" as a reflection of the whole of the condition of the patient that needs an ABT postoperatively.

The outcome of this study can be used for better inform consent, to perform early interventions for prevention of a delirium, to anticipate for a longer LOS and to provide an alternative discharge location early in patients at risk.

Finally, awaiting the results of large trials on ABTs like the FOCUS trial [26], it might be of benefit to use a lower cut-off transfusion threshold in hip fracture patients that are in a worse condition, like anemia.

## Conclusions

This study has demonstrated that anemia at admission and PANT were independent risk factors for worse outcome in hip fracture patients. In multivariable regression analysis, anemia at admission as such had no effect on mortality, due to a rescue effect of PANT. PANT affected in-hospital, 3- and 12-month mortality negatively, with the main effect in the first 3 months postoperatively.

## Competing interests

The authors declare that they have no competing interests.

## Authors' contributions

AV, BB, BM, WT and PP participated in the design of the study. AV, BB, AN, PP and MV were responsible for data collection. BM and WT performed the statistical analysis. PP, RB and RN reviewed the article and supervised the surveillance and analysis. All authors have read and corrected draft versions of the manuscript and have approved the final manuscript.

## Pre-publication history

The pre-publication history for this paper can be accessed here:

http://www.biomedcentral.com/1471-2474/12/262/prepub
